# Ageing-Related Changes in Ultrastructural Bone Matrix Composition and Osteocyte Mechanosensitivity

**DOI:** 10.1007/s11914-025-00927-0

**Published:** 2025-08-12

**Authors:** Yunxin Chen, Jenneke Klein-Nulend, Nathalie Bravenboer

**Affiliations:** 1 Department of Oral Cell Biology, Academic Centre for Dentistry Amsterdam (ACTA), University of Amsterdam and Vrije Universiteit Amsterdam, Amsterdam Movement Sciences, Amsterdam, the Netherlands; 2 Department of Laboratory Science, Amsterdam University Medical Centers (AUMC)/ Location VUmc, Vrije Universiteit Amsterdam, Amsterdam Movement Sciences, De Boelelaan 1117, Amsterdam, 1081 HV The Netherlands

**Keywords:** Ageing, Bone Matrix, Mechanosensitivity, Osteocyte, Ultrastructural composition

## Abstract

**Purpose of Review:**

Bone matrix is a multiscale composite material mainly composed of collagen, crystalline apatite mineral, water, and a small amount of non-collagenous proteins. Nested within bone matrix, mechanosensitive osteocytes orchestrate bone adaptation to mechanical loading, which is affected by the ultrastructural composition and mechanical properties of the osteocyte-surrounding bone matrix. In this review, we shed light on the impact of ageing-related modifications in ultrastructural composition of bone matrix on the mechanosensitivity of osteocytes.

**Recent Findings:**

Ageing modulates the ultrastructural composition of bone matrix, such as collagen cross-links, mineral crystal size, microcracks, content of bound water, content and phosphorylation of non-collagenous proteins, and degree of mineralization. These ageing-related modifications alter the mechanical properties of bone matrix, and the biological function of bone, i.e. altered mechanical properties lead to changes in mechanical loading-induced fluid shear stress experienced by osteocytes, which affect osteocyte mechanosensitivity.

**Summary:**

A better understanding of the role of osteocyte mechanosensitivity in bone adaptation to mechanical loading during ageing is crucial. This review highlights the ageing-related changes in the ultrastructural composition and mechanical properties of bone matrix, that might affect the mechanosensitivity of osteocytes. By linking ageing-related changes in the bone matrix to alterations in osteocyte mechanosensitivity, it is assumed that ageing-modulated bone matrix affects bone adaptation to mechanical loading orchestrated by osteocytes. A comprehensive understanding of how age-related changes in bone matrix composition influence osteocyte mechanosensitivity is crucial for explaining the fragility of ageing bone, as osteocytes are the most abundant and mechanosensitive cells in bone tissue.

## Introduction

Bone matrix is a multiscale composite material mainly composed of collagen, crystalline apatite mineral, water, and a small amount of non-collagenous proteins. Nested within bone matrix, mechanosensitive osteocytes orchestrate bone adaptation to mechanical loading [1]. The osteocytes are characterized by ellipsoid-shaped cell bodies, that reside within lacuna in the mineralized bone matrix. From the osteocyte cell body numerous cell processes sprout through tiny canaliculi forming a connected lacuno-canalicular network [2, 3]. This intricate lacuno-canalicular network serves as a conduit for efficient communication between osteocytes and other (bone) cells [3]. When bone is mechanically loaded, the bone matrix is deformed, which induces a pressure gradient in the interstitial fluid surrounding osteocytes, thereby generating fluid flow in the lacuno-canalicular network [4]. The fluid flow-induced shear stress activates the molecular mechanosensors, such as integrins, primary cilia, calcium channels, and connexin-based gap junctions, on the osteocyte cell membrane [5]. Via mechanical interaction with their niche, osteocytes are subjected to mechanical loads causing deformations of the membrane, cytoskeleton, and/or nucleus [6–9]. As a result, osteocytes release signaling molecules such as nitric oxide and prostaglandin E_2_ [2, 10]. These signaling molecules play a crucial role in the bone adaptive response to mechanical loading [2]. Moveover, bone matrix contains numerous non-collagenous proteins, such as proteoglycans, osteopontin, and osteocalcin, which are related to the properties of bone [11]. Some hormones, e.g., calcitriol, parathyroid hormone, calcitonin, sex hormones (estrogen and testosterone), growth hormones, thyroid hormones, cortisol, and insulin, might be retained in bone matrix and affect bone health in ageing [12]. The role of these non-collagenous proteins in bone is known [13], but whether they are involved in the determination of osteocyte mechanosensitivity remains to be investigated.

The effect of ageing-related modifications of the lacuno-canalicular network on strain-induced fluid shear stress on osteocytes is an important aspect of osteocyte mechanosensation [4]. The transduction of mechanical signals from bone tissue to the osteocytes occurs through the lacuno-canalicular network, but is not covered in this review. Moreover, the impact of ageing-related modifications in the ultrastructural composition of the bone matrix on osteocyte mechanosensitivity is only currently being discovered. The ultrastructural composition of the bone matrix, e.g., collagen cross-links, apatite crystal size, degree of mineralization, microcracks, non-collagenous protein content and phosphorylation degree, and water content in bone are modified during ageing (Table 1). Ageing-related changes in collagen cross-links, apatite crystal size, degree of mineralization, and microcracks are known to be related to changes in the mechanical properties of the bone matrix, e.g., stiffness, thereby affecting the mechanical loading-induced deformation of the bone matrix. The altered deformation of aged bone matrix affects the lacuno-canalicular network deformation, thereby changing the fluid shear stress on the osteocytes. This causes changes in mechanical signaling, which can be sensed by the molecular mechanosensors on the osteocyte cell membrane, and affect osteocyte mechanosensitivity [5]. This review synthesizes recent findings on the ultrastructural composition changes in ageing-modulated bone matrix and explores how these alterations may influence the mechanosensitivity of osteocytes during ageing.

**Table 1:** Ageing-related changes in the ultrastructural composition of bone matrix

Ref #	Species	Ultrastructural component	Increase (+) / decrease (-)
[22–26]	Human	AGEs cross-links	+
[141]	“	Mineral content	-
[47–51]	“	Bone mineral density	-
[77, 141]	“	Microcracks	+
[41]	“	CML	+
[113]	“	GAGs	-
[113]	“	Biglycan	-
[101]	“	Phosphorylation of proteins	-
[101]	“	Phosphorylation of osteopontin	-
[130]	“	Bone water content	-
[129]	“	Bone water concentration	+
[132]	“	Bound water	-
[69]	Rat	AGEs cross-links	+
[70]	“	Crystallinity	+
[70]	“	Mineral-to-matrix ratio	+
[69, 70]	“	Carbonate substitution	+
[69]	“	Pore water	+
[69]	“	Bound water	-
[68]	Mouse	AGEs cross-links	+
[68]	“	Crystallinity	+
[68]	“	Mineral-to-matrix ratio	+
[68]	“	Carbonate substitution	+
[123]	“	Perlecan	-
[68]	“	Bound water	-

CML, Nε-carboxymethyl lysine; GAGs, glycosaminoglycans

## Ageing-Modulated Collagen Fibril Cross-Links and Osteocyte Mechanosensitivity

Collagen type I is abundant in tendon, bone, skin, cornea, lungs, and vasculature [14]. It consists of tropocollagen molecules, which form collagen fibrils [14]. The mechanical properties of collagen are determined by the structure of the cross-linked tropocollagen molecules. The cross-links are considered to be a key component of collagen fibrils as they can change the fibrillar behavior [15]. The enzyme lysyl oxidase mediates the conversion of immature divalent cross-links to mature trivalent cross-links, which are located at the ends of the tropocollagen molecules of collagen type 1 [15, 16]. The density and type of the enzymatic cross-links significantly influence the stiffness of collagen fibrils [17]. Collagen fibrils with trivalent cross-links are stiffer than those with divalent cross-links, at similar cross-link densities [17]. Plastic deformation of human cortical bone has been shown to correlate with collagen maturity [18]. During early life, the content of mature enzymatic cross-links reaches a maximum, after which it stabilizes and remains essentially in the same range throughout adult life [16, 19]. But the enzymatic cross-links alone cannot cause the stiffened regime of bone [20]. This suggests that changes in the content of enzymatic cross-links between tropocollagen molecules is unlikely to be the primary determinant of alterations in the mechanical properties of the bone matrix, and may not affect osteocyte mechanosensitivity during ageing.

Advanced glycation end-product (AGE) cross-links are formed through a non-enzymatic process in bone [21]. They result from the so-called Maillard reaction, which involves the glycation of proteins in the helical regions between tropocollagen molecules. AGE cross-links tend to accumulate over time since collagen has a long half-life (Table 1) [22–26]. An increase in AGE cross-links density modifies the mechanical properties of collagen fibrils at the nano-scale, such as collagen fibril stiffness [27, 28]. At the tissue level, a computational modeling study demonstrated that a high content of AGE cross-links is related to impaired mechanical material properties, e.g., increased brittleness, in aged bone [15]. Stiffer collagen fibrils and brittle bone logically lead to less deformation of bone matrix compared to young bone matrix when subjected to the same force on bone [29]. This will result in reduced fluid shear stress on osteocytes, which affects the mechanosensors in osteocytes, i.e., integrins, which play a crucial role in the detection of mechanical forces and transmission to the cytoskeleton, thereby affecting osteocyte mechanotransduction [8, 9]. Based on the stiffness of the collagen fibrils, the mechanosensitivity of osteocytes may be primarily affected by AGE cross-links accumulation rather than enzymatic cross-links content in collagen fibrils during ageing, since only AGE crosslinks, but not enzymatic cross-links, affect the stiffness of the collagen fibrils.

The crucial roles of the interaction between AGEs and their receptors (RAGEs), such as affecting cell apoptosis, proliferation, and differentiation, which determine cell functionality has been shown in osteoblasts and osteoclasts [30–36]. AGEs and RAGEs interaction may also affect the (osteoblast-derived) osteocytes functionality like mechanosensitivity. AGEs interacting with RAGEs influence bone metabolism and inflammation through the regulation of sclerostin expression in osteocytes [37]. Sclerostin is primarily synthesized by osteocytes and is recognized as a regulator of WNT signaling, influencing the activity of osteoblasts and osteoclasts [38]. Sclerostin has also been shown to exert distant effect, modulating mineral metabolism in the kidney [38]. The mineral of bone is a major determinant of bone mechanical properties [39], which can be affected by mineral metabolism. The AGE content as well as the interaction between AGEs and RAGEs increases with age [40, 41]. This suggest that the change in mineral metabolism and mechanical properties of bone during ageing, ultimately influence mechanosensitivity of osteocytes.

## Ageing-Modulated Bone Mineral and Osteocyte Mechanosensitivity

Human bone mineral is constituted of poorly crystallized apatite, which is calcium (Ca)-deficient apatite containing hydrogen phosphate (HPO_4_), carbonate (CO_3_), and other ions such as minor elements Mg^2+^ or Na^+^, and trace elements Sr^2+^ or F^−^ [42]. The degree of mineralization of bone and the characteristics of the mineral crystallinity are major determinants of bone stiffness and strength [39]. Ageing-related modulation of bone mineral is associated with poor mechanical properties of bone [43, 44]. Moreover, poor mechanical properties of bone are related to osteocyte mechanosensitivity [45, 46]. Therefore, the ageing-related modulation of bone mineral may influence osteocyte mechanosensitivity.

### Mineralization of Bone Matrix– from Tissue To Microstructural Level

The degree of mineralization of bone at the tissue level, also called bone mineral density (BMD), is usually determined by dual-energy X-ray absorptiometry (DXA) to predict bone strength. Ageing is associated with a decreased BMD in the femoral neck [47, 48], and in the hip [49–51] (Table 1), indicating decreased bone strength. The architectural changes in bone during ageing, such as cortical thinning and loss of trabeculae, are mostly responsible for the ageing-related decrease in BMD. A lower BMD at the tissue level affects the mechanical properties of aged human bone, such as elastic deformation [18].

At the microstructural level, the phenomenon of osteocyte lacuna mineralization reflects a decreased number of osteocytes due to cell death and to a lack of newly embedded osteocytes during bone formation [52]. Mineralization in the lacuno-canalicular network increases during ageing [53]. Mineralization of lacunae results in reduced energy-absorbing/dissipating capacity of bone [54]. This makes the bone more brittle, and diminishes octeocyte communication [4]. Changes in mechanical properties lead to changes in deformation of the bone matrix under mechanical loading, resulting in altered mechanical force on osteocytes, which can be detected by integrins [9]. Gap junctions, especially those involving connexin-43, are essential for osteocyte-to-osteocyte communication, enabling the propagation of calcium waves [55]. Thus, ageing-modified bone matrix with poor bone mechanical properties and increased mineralization of osteocyte lacunae may diminish mechanosensitivity via integrins and gap junctions.

### Bone Mineral Composition during Ageing

Mineralized bone functions as a primary reservoir for calcium in the body, with over 95% of the body’s calcium stored in bone tissue [56]. Approximately 65% of the total bone mass is comprised of hydroxyapatite (HA), a crystalline calcium phosphate compound with the chemical formula Ca₁₀(PO₄)₆(OH)₂ [57]. Alternative calcium phases in bone include amorphous calcium phosphate, a transient precursor phase observed in developing bones or sites of rapid mineralization [58]. Amorphous calcium phosphate gradually crystallizes into hydroxyapatite as bone matures [58].

Calcium and phosphorus levels in human blood are regulated by either direct mineral absorbance from the gastrointestinal tract or by mineral mobilization from bone stores by altering bone turnover [58]. Changes in bone turnover cause the release or removal of calcium and phosphorus from bone in a fixed calcium-to-phosphorus (Ca/P) ratio [58]. In ageing bovine cancellous bone, the Ca/P ratio significantly increases from approximately 1.51 in 1-3-month-old bone to around 1.58 in 4-5-year-old bone [59]. Whether the increased Ca/P ratio in ageing bone is driven by elevated calcium or reduced phosphorus concentration in the extracellular fluid of osteocytes remains unclear. The changes in calcium concentration surrounding osteocytes may not necessarily increase or decrease the osteocyte’s ability to sense mechanical stimuli [60]. The number of osteocytes in metatarsal bones of mice responding to three-point bending increases with the magnitude and frequency of loading, albeit the response of a single osteocyte is typically binary (all-or-nothing) [61]. Interestingly, the Ca^2+^ intensity of osteocytes responding to three-point bending did not change significantly with the magnitude of loading, suggesting a threshold for the calcium influx into osteocytes in response to mechanical loading [61]. This threshold may prevent Ca^2+^ cytotoxicity, and suggests that the response to mechanical loading is regulated by the number of osteocytes responding but not Ca^2+^ influx [61]. Moveover, the presence of a calcium gradient on the canalicular wall due to calcium deposition or dissolution generates sufficient fluid flow to induce significant changes in fluid shear stress on the osteocyte membrane [62]. This suggests that calcium fluxes due to ageing-related changes in calcium homeostasis affect osteocyte mechanosensing. T-type voltage-sensitive calcium channels on the osteocyte membrane are uniquely sensitive to fluid shear stress and membrane strain [63]. Primary cilia on osteocytes also sense fluid shear stress and stimulate Ca^2+^ influx [64]. Thus, the calcium surrounding osteocytes may modulate osteocyte mechanosensitivity via T-type voltage-sensitive calcium channels and primary cilia.

### Mineral Crystallinity

Crystallinity of mineral is crucial in the determination of the mechanical properties of human cortical bone [65]. Hydroxyapatite, a crystalline calcium phosphate and the main inorganic constituent in human bone, can readily accommodate substitution(s) by a large variety of ions [66]. Carbonate is a predominant substituent in most biomineral forms of apatite [67]. Carbonate substitution in hydroxyapatite increases with age in bone (Table 1) [68–70]. This increased carbonate substitution reduces mineral crystallinity [65]. Mineral crystallinity also depends on the crystal size of mineral in bone matrix [42]. The crystal size of bone mineral increases with age, resulting in increased mineral crystallinity [71]. Moreover, the mineral crystallinity of human femoral cortical bone improves during the first two decades of life [72]. Thereafter, the mineral crystallinity remains constant and does not change anymore with age [72]. One possible explanation for the maintenance of mineral crystallinity with age is that the increase in crystal size compensates for the effects of elevated carbonate substitution, which typically reduces mineral crystallinity, thereby preserving a consistent crystallinity in the later stages of life. Changes in both mineral crystal size and carbonate substitution in hydroxyapatite affect the mechanical properties of the bone matrix, and are therefore related to osteocyte mechanosensitivity. Remarkably, decreased crystal size with ageing has been reported as well [73, 74]. Differences in reported values of mineral crystallinity are likely the result from the use of varying bone types and sites [71, 73, 74]. Improved understanding of the ultrastructure and mechanical properties of ageing human bone matrix is needed to clarify the relationship between ageing-modulated ultrastructural bone matrix and osteocyte mechanosensitivity. Given the importance of mineral crystallinity for the mechanical properties of bone matrix, ageing-modulated mineral crystallinity may influence osteocyte mechanosensitivity.

## Ageing-Related Bone Matrix Disruption at the Microscale

Microdamage and micropetrosis (lacunar mineralization) may influence the mechanosensitivity of osteocytes by affecting the connection between osteocytes, and between osteocytes and other bone cells, leading to a poor mechanical property of the bone matrix. Bone matrix microdamage is defined as the presence of microcracks as detected using microscopic techniques, e.g. bright-field microscopy, fluorescence (UV light) microscopy, and confocal laser scanning microscopy [75, 76]. Microdamage accumulates with age in weight-bearing bones (Table 1) [77]. The accumulation of microdamage is influenced by the microarchitectural features of lacunae and the surrounding perilacunar zones [78]. Specifically, the presence of lacunae has been shown to increase the risk of microcrack initiation, serving as nucleation sites that accelerate microcrack propagation [78]. Microcracks trigger bone remodeling, but they are not effectively repaired [79]. One possible explanation for insufficient bone repair is osteocyte death caused by microdamage in the bone matrix [75]. Microdamage induces the production of apoptosis-related reactive oxygen species in osteocytes [80]. On the other hand, an ultrastructural study that applied scanning and transmission electron microscopy of mineralized lacunae showed apoptotic remnants of osteocytes within the mineral matter and labeled such lacunae as ‘living fossils’ that preserve the fragments of a previous cell [81]. It is likely that the lacunar mineralization process progresses from osteocyte apoptosis to full lacunar mineralization during ageing [82]. The proportion of micropetrosis to the total lacunar number was less than 2% in young healthy individuals and up to 8% in the older healthy individuals [83]. There may be a causal relationship between age, osteocyte death, and micropetrosis, but this has not been firmly established. Microdamage and micropetrosis contribute to osteocyte death and loss of connectivity between osteocytes [84, 85]. This reduces the osteocyte-to-osteocyte communication via gap junctions, which enable the propagation of calcium waves [55]. Microdamage and micropetrosis also contribute to a decline in the skeletal mechanical properties, including the elastic modulus, fracture toughness, stiffness, and overall strength [86, 87]. A decline in mechanical properties will likely affect the mechanosensitivity of osteocytes by changing the mechanical loading-induced deformation of the bone matrix as well as the fluid flow-induced shear stress on the osteocyte cell membrane, which affects the activation of the mechanosensors in osteocytes, i.e., integrins [9], and primary cilia [88]. This suggests that osteocyte mechanosensitivity diminishes during ageing.

## Non-Collagenous Proteins in Bone Matrix and Osteocyte Mechanosensitivity

Non-collagenous proteins play essential roles in regulating mineralization and crystallinity of the bone matrix. Since mineralization and bone crystallinity are important in the determination of the mechanical properties of bone, ageing-related alterations in concentration, composition, and distribution of non-collagenous proteins within the matrix may influence osteocyte mechanosensitivity. Non-collagenous proteins primarily consist of glycoproteins, gamma-carboxyglutamic acid (Gla)-containing proteins, proteoglycans, growth factors, and cytokines, and comprise approximately 10% of the organic phase of the bone matrix [13, 89–94].

### Glycoproteins

Glycoproteins are notable for their site-specific binding to collagen [95]. They accumulate and/or interact with calcium ions in the bone matrix, thereby modulating crystal nucleation and growth [96]. This affects mineralization of bone matrix. Therefore, glycoproteins may influence osteocyte mechanosensitivity by regulating the mechanical properties of bone matrix. Glycoproteins are characterized by a covalent linkage of sugar moieties attached via asparaginyl or serinyl residues [97]. They are modified through phosphorylation [13]. Osteopontin (OPN), bone sialoprotein (BSP), and dentin matrix protein-1 (DMP-1) are the small integrin-binding ligand with N-glycosylation (SIBLING) glycoproteins in bone [96]. Osteopontin is the most extensively studied phosphoprotein [98]. It exerts dual effects on hydroxyapatite formation, depending on its phosphorylation level [99, 100]. Low phosphorylation of osteopontin inhibits, whereas high phosphorylation promotes hydroxyapatite formation [100]. Ageing is associated with a decline in the overall phosphorylation level of non-collagenous proteins, such as reduced (~ 30%) osteopontin phosphorylation in the ninth decade of human life (Table 1) [101]. Declined osteopontin phosphorylation alters hydroxyapatite formation in the bone matrix, potentially affecting the mechanical properties of bone, thus affecting osteocyte mechanosensitivity.

### Bone-Gla-Containing Proteins

Ageing-modulated changes of Bone-Gla-containing proteins (BGP, or osteocalcin) may be related to osteocyte mechanosensitivity as osteocalcin plays a role in bone mineralization and remodeling. Osteocalcin, a 49 residue water soluble protein synthesized in calcified tissues, constitutes roughly 20% of the non-collagenous proteins in bone [102, 103]. Osteocalcin undergoes post-translational modification by vitamin K-dependent enzymes to produce gamma-carboxyglutamic acid (Gla) [102]. Osteocalcin contributes to bone mineralization by binding with high affinity to bone mineral causing an acceleration of hydroxyapatite nucleation [103, 104]. It influences osteoclast recruitment and differentiation, thereby affecting bone resorption [105, 106]. It affects osteoblast recruitment thereby influencing bone formation [107, 108]. The distribution patterns of osteocalcin in human bone change with age [109]. Within individual osteons, four different distribution patterns of osteocalcin are arbitrarily defined [109]. Moreover, osteoblasts in old people produce less osteocalcin during their lifespan in comparison with those in young people [110]. Additionally, newly formed osteonal bone contains more osteocalcin then older interstitial bone, also suggesting that the amount of osteocalcin in bone is related to the age of the bone [11]. The question remains whether osteocalcin distribution content and distribution affect the mechanical properties of bone matrix.

### Proteoglycans

Proteoglycans have been shown to influence the mechanical behavior of bone [111–113]. Ageing-related changes may therefore affect the mechanosensitivity of osteocytes embedded in bone matrix in response to mechanical loading. Proteoglycans are characterized by the covalent attachment of long-chain polysaccharides (glycosaminoglycans, GAGs) to a core protein molecule. Proteoglycans affect collagen fibrillogenesis and matrix mineralization [114, 115]. Ageing-related changes in bone proteoglycans include decreased chondroitin sulfate (CS), a major glycosaminoglycan subtype, and biglycan, a small leucine-rich repeat proteoglycan (Table 1) [113]. Loss of glycosaminoglycans reduces the toughness of bone at the tissue level [116]. Perlecan, a heparan sulfate proteoglycan encoded by the *hspg2* gene, features five globular domains and 3 or 4 attached glycosaminoglycan side-chains [117]. It is present in bone matrix [118]. It is a key component of the osteocyte pericellular matrix [119]. Perlecan interacts with cell-surface receptors, matrix molecules, and growth factors [120, 121]. Moreover, perlecan has been suggested to serve as a tethering element and barrier molecule within the pericellular space in the lacuno-canalicular network, facilitating nutrient exchange and cellular communication [122]. The density of perlecan declines with age [123]. Perlecan deficiency is associated with an impaired bone response to mechanical stimuli [123, 124], accelerated bone mineralization, narrowed lacuno-canalicular network channels [122], and impaired calcium signaling in osteocytes [125]. Perlecan therefore might act as sensing antenna of osteocytes within the space of the lacuno-canalicular network influencing osteocyte mechanosensitivity during ageing.

## Water in Bone Matrix and Osteocyte Mechanosensitivity

Water is a significant component of the bone matrix, distributed across two distinct compartments: pore water and bound water [126]. The volume of pore water residing within intracortical porosity corresponds to the spaces within the bone matrix, predominantly formed by Haversian canals and the lacuno-canalicular network [126]. Bone water content has been shown to correlate with the mechanical properties of the bone matrix [126–128]. Additionally, the movement of water within the lacuno-canalicular network in response to pressure gradients generated during mechanical loading affect osteocyte mechanosensitivity. During ageing the water concentration in human femurs increases [129]. Pore water within the Haversian canals and lacuno-canalicular network also increases with age [69, 126]. The increase in water concentration should be considered independently with the increase of bone porosity with age [126]. The bone water content has been reported to decrease within bone tissues with age [130]. This may stem from differences in sex, sample sources, donor demographics, and analytical techniques to measure water content in bone (Table 2), highlighting the need for further investigations, particularly in human bone.

**Table 2:** Ageing-related changes of bone water

Ref #	Species	*N*	Gender, ♂/♀/♂♀	Age range, y/mo	Bone type	Method	Bone water, increase (+) / decrease (-)
[130]	Human	7	♀	26–82 y	Calcaneous	TRS	Water content, +
[132]	Human	18	♂	47–87 y	Femur	NMR	Bound water, +
[138]	Human	14	♂♀	21–99 y	Cortical	NMR	Bound water, - (shift: loosely to tightly)
[129]	Human	72	♂♀	20–80 y	Tibia	MR	Bone water conc., +
[69]	Rat	12	♂	6,12,24 mo	Femur	NMR	Bound water, - Pore water, +
[68]	Mouse	≥ 11	♂♀	6,20 mo	Femur	NMR	Bound water, -

N, sample size; ♂/♀/♂♀, male/female/male and female; y/mo, years/months; TRS, time-resolved transmittance spectroscopy; MR, hybrid radial ultrashort echo time magnetic resonance; NMR, nuclear magnetic resonance

Bound water, another water compartment in bone, plays a critical role in bone’s mechanical behavior, as it is essential for imparting ductility or plasticity to collagen [126]. Bound water within the bone matrix arises from hydrogen bonding with collagen and electrostatic attractions with minerals, exhibiting varying degrees of affinity [126, 131]. The bound water content decreases with age in bone [68, 69, 132]. A decreased bound water content has been attributed in part to the higher stability of collagen fiber networks due to increased AGE cross-links within the ageing-modulated bone matrix, potentially hindering water binding [133–135]. The loss of proteoglycans and glycosaminoglycans in bone matrix contributes to this decline, since these molecules, being polar and highly negatively charged, have the capacity to attract water into the matrix, e.g., in articular cartilage [136]. Loss of glycosaminoglycans is associated with a significant reduction in the toughness of bone at the tissue-level, which is mediated by the associated loss of bound water [116, 137, 138]. The ageing-related shift from lower-energy to higher-energy interactions between water and solid protons in the bone matrix also contributes to the reduction in bone matrix toughness [138]. Ageing-related changes in bound water in bone leads to alterations in the mechanical properties of the bone matrix [126]. These ageing-modulated mechanical properties affect the function of the mechanosensors in osteocytes, e.g., integrins, which play a crucial role in the detection of mechanical forces and their transmission to the cytoskeleton [9]. This might affect the mechanosensitivity of osteocytes as such that it ultimately causes bone fragility during ageing.

## Do Ageing-Related (Ultrastructural) Differences in Bone Matrix Affect Osteocyte Mechanosensitivity?

**Fig. 1:**
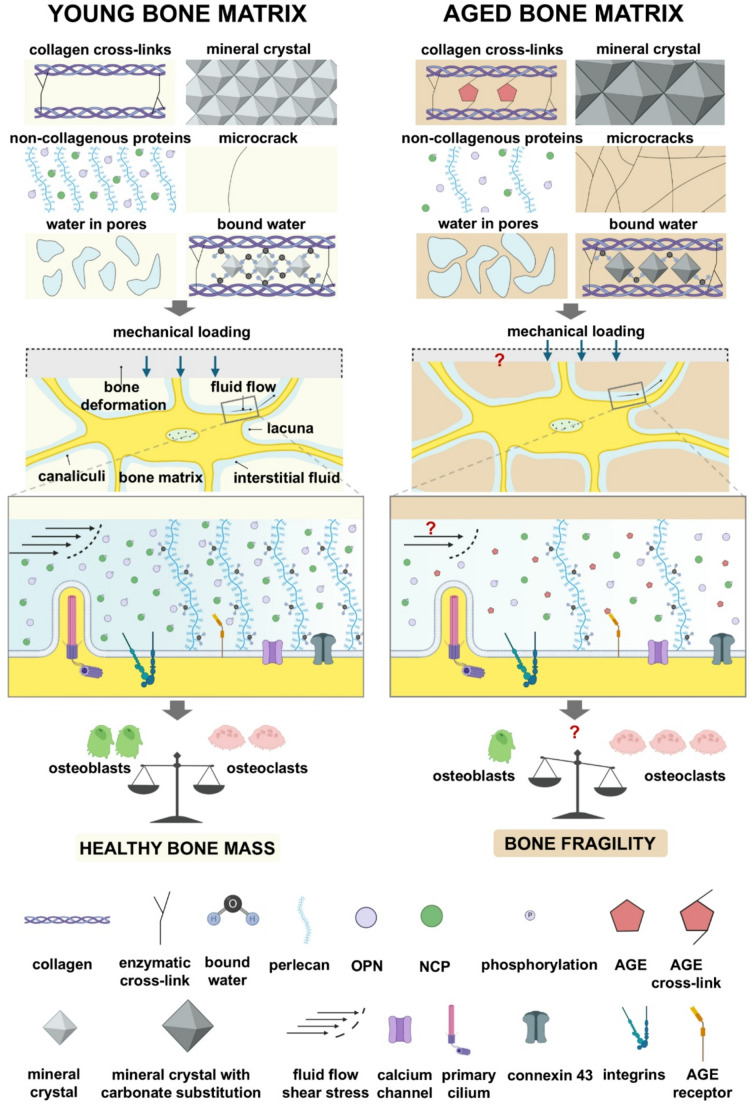
Ageing-related changes in bone matrix ultrastructural composition and their impact on mechanical properties and osteocyte mechanosensitivity. With age, the accumulation of advanced glycation end products (AGEs) in the bone matrix increases collagen fibril stiffness and bone brittleness. With age, the size of mineral crystals in the bone matrix also increases, as well as the extent of carbonate substitution in calcium phosphate crystals, affecting the crystallinity of bone matrix. Ageing-related decline in non-collagenous protein phosphorylation, e.g., osteopontin, impairs hydroxyapatite formation, further affecting bone mechanics. The density of perlecan, a crucial barrier molecule in the lacuno-canalicular network, decreases with age, thereby affecting the bone’s response to mechanical loading. Accumulating microcracks are not effectively repaired in aged bone matrix, contributing to reduced mechanical strength. Pore water in the bone matrix increases with age, correlating with increased space within bone matrix. Bound water within the bone matrix arises from hydrogen bonding with collagen and electrostatic attractions with mineral crystals. The bound water content decreases with age, affecting the stability of collagen fibers. Together, these ultrastructural changes in the aged bone matrix may lead to altered mechanical properties. For instance, the aged bone matrix is stiffer than the young bone matrix, which may result in less bone deformation under mechanical loading. This reduced deformation leads to a decrease in fluid flow within the lacuno-canalicular network of osteocytes. Molecular mechanosensors, such as integrins, primary cilium, calcium channels, connexin 43, and perlecan, can detect changes in fluid flow within the lacuno-canalicular network. Moreover, AGE interact with the receptor for advanced glycation end products (RAGEs) in osteocytes, potentially affecting bone remodeling. Consequently, osteocytes embedded in the aged bone matrix may respond differently compared to those embedded in the young bone matrix. This differential response may regulate bone remodeling, influencing osteoblastic bone formation and osteoclastic bone resorption, ultimately contributing to the fragility of ageing bone. OPN, osteopontin. NCP, non-collagenous protein. Figure created using BioRender

Ageing-related ultrastructural changes in the bone matrix include alterations in collagen cross-links, bone mineralization, non-collagenous proteins, and water content, which modulate the mechanical properties of bone. The changes in mechanical properties in turn may affect the mechanical loading-induced fluid shear stress on the osteocyte processes. The ageing-related modifications of the mechanical properties of bone may impair osteocyte mechanosensation and mechanotransduction (Fig. 1). The changes in bone matrix mechanics and fluid flow-induced shear stress affect osteocytes by activating the molecular mechanosensors, e.g., integrins, primary cilia, calcium channels, and connexin-based gap junctions, on the osteocyte cell membrane [5]. Integrins in osteocytes play a crucial role in the detection of mechanical forces, i.e., fluid shear stress, and in the transmission of mechanical forces to the cytoskeleton [9]. Ageing-related ultrastructural changes of the bone matrix lead to poor mechanical properties and possibly less deformation of the bone matrix, which affects the detection of the mechanical forces sensed by integrins in osteocytes. Primary cilia on the osteocyte cell body, but not on the cell processes, sense mechanical loading-induced fluid shear stress, and contribute to the biochemical response of osteocytes in their native matrix [88]. Ageing increases the number of mineralized osteocyte lacunae [53]. This may reduce fluid shear stress on osteocytes in ageing bone matrix, that subsequently would limit the stimulation of primary cilia and reduce osteocyte mechanosensation. T-type voltage-sensitive calcium channels are particularly responsive to membrane strain and fluid shear stress induced by a calcium gradient generated on the canalicular wall [62, 63]. Gap junctions, especially those involving connexin-43, are essential for osteocyte-to-osteocyte communication [139]. Ageing-induced increased microdamage or micropetrosis diminishes osteocyte communication [53, 77], thereby reducing osteocyte mechanosensation in ageing [139]. Osteocytes may experience a decline in their ability to sense and adapt to mechanical forces due to ultrastructural matrix changes, which may negatively impact bone health as age progresses (Fig. 1).

## Summary

The AGE cross-link content, AGEs concentration, mineralization, microcracks accumulation, Ca/P ratio, carbonate substitution, non-collagenous peoteins phosphorylation, non-collagenous protein distribution, non-collagenous peotein expression, and bone water content change with age in bone matrix. The ageing-modulated ultrastructural composition changes the mechanical properties of the bone matrix. Ageing-related differences in the ultrastructural composition of the bone matrix likely affect osteocyte mechanosensitivity.

Ageing-related changes in the ultrastructural composition of the human bone matrix are the base of understanding osteocyte mechanosensitivity during ageing. With the advancement of techniques, such as nano-scale measurements of bone matrix, more detailed information on ageing-related changes of bone mineral composition, mineral crystallinity, and non-collagenous protein composition is expected. However, whether and how ageing bone matrix, in particular the ultrastructural composition, affects osteocyte mechanosensitivity is still largely unknown. To address this research gap, a three-dimensional mechanical loading model of human osteocytes in their native matrix is needed to investigate mechanosensation in different age groups. Such a model using bone explant tissue has been developed [140]. Using this model, the mechanosensitivity of osteocytes and ageing-related changes in osteocyte mechanosensitivity can be detected [140]. Moreover, it is also possible to measure the mechanosensitivity of osteocytes isolated from their native bone matrix by measuring nitric oxide production by osteocytes in response to mechanical loading [92]. Furthermore, detailed information of the ultrastructural composition of bone matrix, and the ageing-related changes could serve as an entrance for finite element models, predicting the deformation of bone matrix under mechanical loading. Using finite element modeling to predict the deformation of bone matrix will provide more accurate data on the relationship between the ultrastructural composition, mechanical properties, and altered fluid shear stress under mechanical loading. This may contribute to a better clarification of the relationship between the ageing bone matrix and osteocyte mechanosensitivity.

In conclusion, ageing-modulated ultrastructure and composition of bone matrix affect the mechanosensitivity of osteocytes by altering mechanical and chemical signaling in the osteocyte sensing environment. Especially ageing-modulated non-collagenous proteins seem to play an important role in determining the ageing-related changes in osteocyte mechanosensitivity. Understanding the relationship between ageing-modulated bone matrix and osteocyte mechanosensitivity will help to better understand the cause of bone fragility in ageing populations. This knowledge could help to develop targeted therapeutic strategies to prevent or mitigate bone fragility and improve bone health in the ageing population.

## Data Availability

No datasets were generated or analysed during the current study.
